# Communicating with patients with nAMD and their families during the COVID-19 pandemic

**DOI:** 10.1007/s00417-020-04697-6

**Published:** 2020-04-22

**Authors:** Jean-François Korobelnik, Anat Loewenstein, on behalf of the Vision Academy, Tariq Aslam, Jane Barratt, Bora Eldem, Robert Finger, Richard Gale, Monica Lövestam-Adrian, Mali Okada, Nick Parker, Francisco Rodriguez, Michelle Sylvanowicz, James Talks, Tien Yin Wong

**Affiliations:** 1 Service d’ophtalmologie, CHU Bordeaux, Bordeaux, France; 2 Inserm, Bordeaux Population Health Research Center, team LEHA, Université de Bordeaux, UMR 1219, F-33000 Bordeaux, France; 3 Division of Ophthalmology, Tel Aviv Medical Center, Sackler Faculty of Medicine, Tel Aviv University, Tel Aviv, Israel

Dear Editor,

In discussion with colleagues around the world, we have identified a critical gap of guidance for clinicians to communicate with patients and their families about how to minimize exposure whilst undertaking crucial eye care services during the current COVID-19 pandemic.

In the current environment, patients with neovascular age-related macular degeneration (nAMD) and their families are concerned and anxious about attending regular appointments to maintain their vision due to the risk of exposure.

The safety and well-being of our patients are of the utmost importance. The impact of a recurring treatment schedule during this pandemic, where there are severe restrictions on clinical services and social distancing measures, is among the important aspects to be addressed.

Firstly, a clear explanation of infection prevention protocols and safeguards of each clinic, including what to expect before, during, and after the appointment, may help to alleviate concerns.

We assessed international retinal practices and noted a lack of consistent evidence-based guidelines or readily available information for clinicians to communicate to their patients on how their clinics are adapting in response to COVID-19.

We at the Vision Academy [1], with support from Bayer, have compiled a guidance document that explains how to adapt clinic practices to minimize risk of exposure of patients and medical staff, and to prioritize those with the greatest treatment need [2]. The suggestions are based on the latest clinical recommendations [3], which can be adapted to local regulations and standards. In addition, we have developed a communication template [4] that can be used as a proactive tool, to be sent via email or text message, to patients and their families ahead of their appointment to reassure them that their safety and eye health remains a priority.

A shortened version of the guidance document is provided below (full version available elsewhere [2]).

Informing patients and families about what to expect at the next appointment

- Before the appointment, the clinic may reach out to inquire about the patient’s current health status.
- The clinic schedule may be adjusted to permit the minimum number of people in the waiting room at any given time.
- Regular visual acuity testing or eye scans before an anti-VEGF procedure may not be required to minimize the time spent in the clinic.
- The ophthalmologist may wear a mask with a plastic shield over their eyes and limit conversation.
- To limit exposure, scheduling of the next appointment may be via phone rather than in the clinic.

Educating patients on how to reduce the risk of exposure during and between clinic visits

- Ask patients to notify your clinic ahead of the appointment if they have had direct exposure to a COVID-19-positive person, have a cough/fever or other symptoms indicative of exposure, and reschedule the appointment.
- If the patient is feeling unwell, propose that their appointment be rescheduled.
- Request patients to attend the appointment with only one companion who may have to wait outside the clinic to comply with social distancing protocols.
- Instruct patients to maintain a distance of at least 2 m (6 ft) whilst in the waiting room.
- Patients may be given a mask to wear during treatment.
- In case of cancellation, ask patients to reschedule as soon as able.
- Prior to the next scheduled appointment, ask patients to regularly monitor their vision with an Amsler Grid test, alternating eyes when conducting the test.
- Encourage patients to contact the clinic if they notice a change in their vision to assess if an emergency visit is needed.

We hope that these documents are a useful resource to the medical community.

## Contributing authors

Prof. Tariq Aslam

Consultant Ophthalmologist

Manchester Royal Eye Hospital

UK

Dr. Jane Barratt

Secretary General

International Federation on Ageing

Canada

Prof. Bora Eldem

Professor of Ophthalmology

Hacettepe University

Turkey

Prof. Robert Finger

Professor of Ophthalmic Epidemiology and Retina Consultant

University of Bonn

Germany

Prof. Richard Gale

Consultant Medical Ophthalmologist and Clinical Director in Ophthalmology

Honorary Visiting Professor

University of York

UK

Dr. Monica Lövestam-Adrian

Head of the Medical Retina Department

Lund University Hospital

Associate Professor in Ophthalmology

Lund University

Sweden

Dr. Mali Okada

Royal Victorian Eye and Ear Hospital

Australia

Mr. Nick Parker

International Agency for Prevention of Blindness

UK

Dr. Francisco Rodriguez

Scientific Director

Fundación Oftalmológica Nacional

Chair of the Department of Ophthalmology

Universidad del Rosario School of Medicine

Colombia

Ms. Michelle Sylvanowicz

Director of Global Advocacy

Bayer

Switzerland

Mr. James Talks

Consultant Ophthalmologist

Royal Victoria Infirmary

UK

Prof. Tien Yin Wong

Medical Director

Singapore National Eye Centre

Provost’s Chair Professor of Ophthalmology

Duke-NUS Graduate Medical School

Chair of the Singapore Eye Research Institute

Singapore
